# Gift to University College Hospital

**Published:** 1924-06

**Authors:** J. Gerald Buckle

**Affiliations:** Secretary. University College Hospital, W.C. 1


### Gift to University College Hospital.

Sir,—With reference to the paragraph in your last issue, may I point out that Mr. Geoffrey Duveen's gift of £15,000 to University College Hospital is for the purpose of providing "the most complete and modern treatment of the deaf," and is not intended for the general purposes of the hospital? The money will be expended entirely on the Ear, Nose and Throat Department, and the removal of the Royal Ear Hospital (Ear, Nose and Throat Department of University College Hospital) from its present site in Dean Street to a site in the neighbourhood of University College Hospital is in contemplation.

J. Gerald Buckle,
University College Hospital, W.C. 1. Secretary.

---

**A Novel Scheme for Raising Money.**

The latest scheme for raising money for the London hospitals is a series of conducted tours to places of historic interest in the metropolis, which King Edward's Hospital Fund has arranged. The guides, who give their services, are authorities upon the places visited, such as St. James' Palace (the first of the series), the Tower of London, the Mint, the Temple, Somerset House, the Houses of Parliament and the Abbey. Single or season tickets can be obtained.

